# “It’s not necessarily science based”: understanding organ utilisation decision-making in cardiothoracic transplantation in the UK through qualitative interviews

**DOI:** 10.1136/bmjopen-2026-121139

**Published:** 2026-10-05

**Authors:** Rebeka Jenkins, Gillian Hardman, Karen Booth, John Dark, Andrew Fisher, Catherine Exley

**Affiliations:** 1 Population Health Sciences Institute, Faculty of Medical Sciences, Newcastle University, Newcastle upon Tyne, UK; 2 Statistics and Clinical Studies, NHS Blood and Transplant, Bristol, UK; 3 Freeman Hospital, Newcastle upon Tyne Hospitals NHS Foundation Trust, Newcastle upon Tyne, UK; 4 Translational and Clinical Research Institute, Faculty of Medical Sciences, Newcastle University, Newcastle upon Tyne, UK

**Keywords:** Clinical Decision-Making, TRANSPLANT SURGERY, QUALITATIVE RESEARCH, Cardiothoracic surgery

## Abstract

**Abstract:**

**Objectives:**

This study explores decision-making in organ transplantation. The number of patients waiting for a solid organ transplant continues to rise despite efforts to increase the available pool of donor organs. This has focused attention on maximising the number of organs offered that are subsequently transplanted, a process termed organ utilisation. There is a paucity of work exploring how clinicians decide to decline or accept offers of donor organs. This study aims to better understand the factors that influence organ utilisation decision-making in cardiac and thoracic transplantation through qualitative methods.

**Design:**

Semi-structured qualitative interviews undertaken online over a 5-month period. Data collection and analysis occurred simultaneously. Transcripts were inductively analysed to generate themes.

**Setting:**

Clinicians representing all six adult UK cardiothoracic transplant centres.

**Participants:**

Consultant cardiothoracic transplantation surgeons (n=9) and cardiothoracic transplant coordinators (n=10).

**Results:**

19 interviews were undertaken. The following themes pertinent to utilisation decision-making were identified: *anticipation of outcomes; data; motivation; out-of-hours; micro-culture* and *experiential learning*.

**Conclusions:**

This study is the first to explore the process of organ utilisation decision-making from a clinician’s perspective through interviews. This documents an important and nuanced understanding of the interrelated factors involved in utilisation decision-making in practice. By providing evidence for the human inconsistencies in utilisation decision-making, this work may inform a more considered approach to strategies to improve utilisation rates.

**STRENGTHS AND LIMITATIONS OF THIS STUDY**

- Interviews were conducted with the clinicians most closely involved in organ utilisation decisions in heart and lung transplantation.
- Participants were consultant cardiothoracic transplant surgeons and cardiothoracic recipient coordinators.
- All UK adult heart and lung transplanting centres were represented.
- Interviews captured rich qualitative data on organ utilisation decision-making.
- Interviews are a retrospective view and do not include the complete multidisciplinary team.

## Introduction

 Solid organ transplantation is a life-saving treatment option for patients with organ failure.^1^ There is, however, an ongoing challenge with acquiring enough organs for all eligible patients^2^ and there is an increasing list of patients awaiting transplantation.^3^ There have been efforts to improve the pool of available donated organs, with concerted campaigns to increase the public’s awareness of organ donation.^4^ Studies have demonstrated that organs can be safely used from a wider range of donors, including hearts from donors after circulatory death,^5^ hearts from older donors^6^ and lungs from donors who smoked.^7^ Despite this, there is evidence that suitable organs are not being transplanted, for example, lungs from older donors^8^ and organs considered to be of higher quality.^9^ This has turned the attention of the transplant community to understanding how best to maximise organ utilisation.^9–11^ Organ utilisation is the term given to the process of accepting an organ from a potential donor and subsequently implanting it into a recipient.^12^ Organ utilisation in heart and lung varies internationally,^13^ and particularly in the UK there are overall relatively low organ utilisation rates for heart and lung(s) from consented donors.^9 10^ There is acknowledged to be variation in organ utilisation between transplant centres and individual clinicians; this is observed in practice,^14 15^ in clinical trials^16^ and demonstrated in response to simulated offers.^17 18^ Why such variation in organ utilisation exists is not fully understood^11^ but the question has become a matter of increasing public interest.^19 20^

The progression of organs from offer to eventual implantation involves a sequence of steps.^9^ Organ offering and distribution processes in the UK are complex and tightly regulated. In the National Health Service (NHS), adults potentially suitable for heart or lung transplantation are assessed and registered according to selection policies and assigned one of three urgency categories.^21 22^ Offering processes for hearts and lungs proceed according to automated algorithms designed to maximise equity of access and accounting for the varied urgency of prospective recipients.^23 24^

In contrast to centralised and standardised offering processes, the decision to accept an organ offered for transplantation rests with the individual transplanting team and specifically with the transplanting surgeon. In the UK, specialist trained cardiothoracic recipient nurse coordinators receive details of an organ offer from a centralised service. They then collate the available information regarding the offer, which is then discussed with the on-call consultant cardiothoracic transplant surgeon. It is the surgeon who makes the final decision to accept or decline the offer supported by the coordinator. In practice, transplanting teams may be asked to consider an organ offered for a specific named recipient, or to select from a list of prospective eligible candidates on the non-urgent waiting list at their centre.^25 26^

Within the organ utilisation pathway, the ultimate decision to accept or decline an offered organ remains a human-driven process. Clinical decision-making is acknowledged to be complex and influenced by human behaviour and social and contextual factors.^27 28^ The work to explore these factors in the field of organ utilisation is lacking, however.^29^ To date, studies of decision-making in transplantation have centred on organ donation^30^ and shared decision-making with patients.^31^ Work regarding organ utilisation decision-making by transplanting clinicians remains largely theoretical, for example, applying theoretical models from the field of behavioural economics.^32–34^ There remains a lack of empirical qualitative evidence of how organ utilisation decisions are made in clinical practice.^35^ A greater understanding of the factors that influence the transplanting surgeon’s decision to accept or decline a donor organ is needed—including the supporting role of the transplant coordinator. This has important implications for the strategies to improve organ utilisation.

This paper aims to critically examine utilisation decision-making in practice in UK heart and lung transplantation. It harnesses the advantages of qualitative methods to drive understanding of how complex behaviours are enacted in a real-world transplantation setting.^36^ By exploring first-hand accounts from clinicians most closely involved in organ utilisation decisions for hearts and lungs, this work aims to deepen understanding of how surgeons describe deciding which donor organs to accept for transplantation supported by transplant coordinators, and how decision-making practice is learnt and refined over time.

## Methods

### Study design

In this qualitative study, data were collected through semi-structured interviews. The analysis drew on a constant comparison method derived from constructivist grounded theory.^37^ This supported an inductive interpretivist approach to data analysis where there is little existing evidence, allowed for multiple perspectives to be simultaneously acknowledged and for emerging lines of analytical inquiry to be explored in future interviews. The analytical approach incorporated ideas of interpretivism and collective idealism, considering how an understanding of the world is shared and communicated by groups in particular contexts.^38^ Interviews were undertaken and analysed by a senior cardiothoracic surgical trainee and clinical research fellow at NHS Blood and Transplant (GH, female, BSc MBBS MSc), who used reflection and group discussion to combine clinician and social scientist perspectives in the analysis process. Qualitative work allows a richly detailed exploration of the subtle and interacting factors influencing organ utilisation. The result is an account of the nuanced real-world understanding of organ utilisation decision-making by clinicians in the UK cardiothoracic transplantation programme. The study is reported in accordance with qualitative reporting guidelines^39^ (see also online supplemental file I).

### Participant selection and setting

Eligible participants were consultant adult heart and/or lung transplant surgeons, and cardiothoracic recipient coordinators currently working in UK transplant centres. Invitations to participate were circulated to surgeon and coordinator representatives in each of six adult UK cardiothoracic centres identified through the NHS Blood and Transplant Cardiothoracic Transplant Advisory Group.^40^ They were asked to disseminate invitations to participate to all potentially eligible clinicians within their centre. Expressions of interest were registered via email with the primary investigator (GH). No information was collected on those declining to participate. The primary investigator latterly approached individuals directly to support purposive sampling. Participants were purposively sampled for a range of (1) transplant centre, (2) surgeon or coordinator role and (3) length of time in current post. Participants provided written consent prior to the interview and confirmed verbally at the start of the interview. The study received appropriate ethical approval.

### Data collection and analysis

Semi-structured interviews were conducted via online video conferencing between 1 March and 31 July 2022 by GH, a cardiothoracic surgery trainee (female, MSc, Clinical Research Fellow). One-time interviews followed a predefined and piloted interview guide (see online supplemental file II). Interviews were recorded, transcribed verbatim and anonymised, only the interviewer and participant were declared present and transcripts were not returned to participants. Data collection and analysis occurred simultaneously, supported by NVivo qualitative data analysis software (QSR International (2020)) and the interviewer’s field notes made following each interview. Coding was undertaken by GH and discussed with the wider team which included cardiothoracic transplantation clinicians and an applied health academic. Line-by-line inductive coding of the first five anonymised transcripts (one surgeon and four coordinators) identified, described and analysed patterns derived directly from the interviews. This shaped the interview guide for subsequent interviews. Through iterative rounds of analysis more defined themes were developed, latterly using a deductive approach to test and refine themes. Rather than a traditional assessment of data saturation, it was judged that sufficiently rich data from experts with direct lived experience (high information power) had been collected to answer the study’s specific research question^41^ after 19 interviews—whereafter data collection ceased.

### Patient and public involvement

The need to explore the unwarranted variation in acceptance practice was developed from the co-produced recommendations from the UK’s Organ Utilisation Group which included extensive patient and public involvement.^10 12^

## Results

19 semi-structured interviews were conducted, with 9 consultant cardiothoracic transplant surgeons and 10 cardiothoracic recipient coordinators. Participant characteristics are outlined in table 1. Interviews lasted between 30 and 80 min (mean 55 min). Each UK cardiothoracic transplanting centre was represented by at least one surgeon and one coordinator.

**Table 1:** Characteristics of interview participants

Characteristic		N
Consultant surgeon participants		9
Gender	Male	9
Years in role	<5 years	3
	5–10 years	3
	>10 years	3
Transplant coordinator participants		10
Gender	Female	7
	Male	3
Years in role	<5 years	3
	5–10 years	4
	>10 years	4

Analysis of interview transcripts identified six themes. These are: *anticipation of outcomes* for prospective organ recipient(s)*; data* (availability, reliability and trust in information available at the time of organ offering); *out-of-hours* working*; motivation* (positive and negative external and intrinsic drivers to transplant); *microculture* (between individuals in working relationships, within a team, a department and wider local and national communities); and *experiential learning* (the process of skills acquisition and development). These themes, and their relationships to each other, are summarised in figure 1.

**Figure 1:**
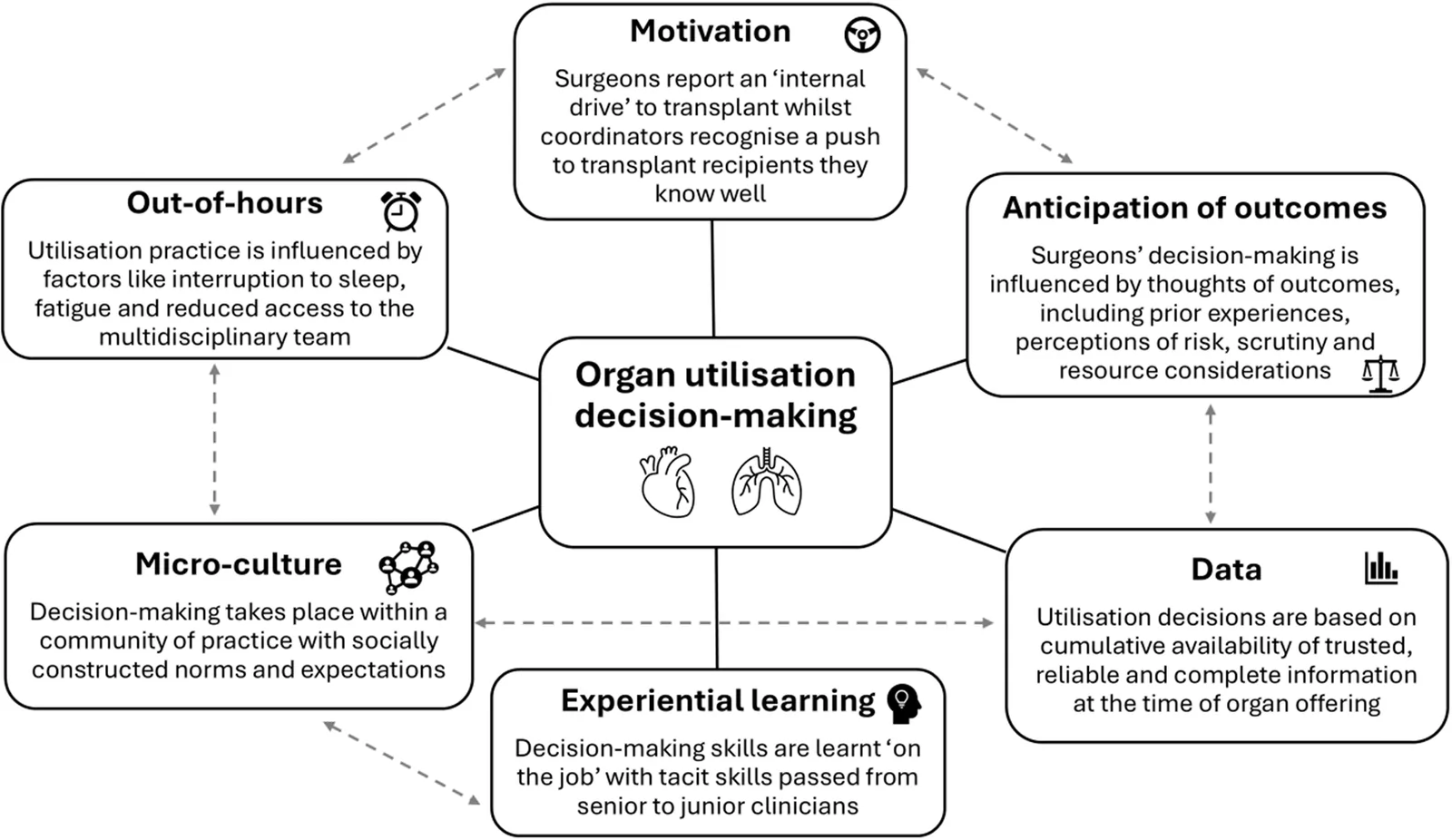
Summary of findings from qualitative interviews with cardiothoracic transplant surgeons and transplant coordinators exploring organ utilisation decision-making

### Anticipation of outcomes

*Anticipation of outcomes* encompassed predicting the result of proceeding with transplantation after appraising a set of donor characteristics, and the chance of a potential recipient dying awaiting transplant if the offer is declined. Prevalent in all participants’ accounts of decision-making was that beneficence was a foundational principle underpinning decision-making.

I think, for the vast majority [of clinicians] your reason for doing an operation, whatever the operation, is that you believe it is the right thing to do for the patient. You are doing a good service to the patient, whether it’s as simple as prognosis or whatever outcome measure, you are doing it because you think it is the right thing for the patient - Consultant Surgeon 02

Reference was made to specific donor characteristics that most heavily influence decision-making. Surgeon participants in particular noted traditional ideal donor characteristics that positively influence their decision to accept.

Generally, the younger the donor the better. If you have a borderline organ in a younger donor you are probably still going to get better results. So if I had to say one thing I look at its donor age and the younger donor - Consultant Surgeon 04

*Anticipation of outcomes* also required detailed knowledge of recipients’ circumstances and judging the risk of dying awaiting a transplant if transplantation did not proceed at that time. This was emphasised by coordinator participants especially.

I think with urgent patients, you know actually whatever comes along is probably better than what they have, so you will accept more risk and a borderline donor. With routine patients, they have more time, then you tend to be more conservative, in stable patients perhaps - Consultant Surgeon 03

Decision-making was noted to be even more complex when an offer is made that requires the transplanting centre themselves to select a suitable recipient from a list of prospective recipients. Participants described making a balanced judgement of risks that prioritised the individual perceived to be most in need.

So, if you’ve got somebody with COPD [chronic obstructive pulmonary disease]) who was oxygen dependent and an IPF [Idiopathic pulmonary fibrosis]), I would, well, depending on the patient, because we do have some sick COPDs, but generally, I would choose the IPF, be more willing or wanting to get them transplanted. We are lucky, we know our waiting list really well. We can see how long they’ve been waiting, what is their trajectory - Recipient Coordinator 09

The potential complexity of the surgical procedure was also reported to be a factor when selecting a recipient. Some transplant coordinators perceived that surgeon colleagues selected recipients based on a lower chance of technical complications.

Some surgeons, where I’ve offered them organs, they will pick the DCM [dilated cardiomyopathy]) over the VAD [ventricular assist device] explant, because its less complicated - Recipient Coordinator 07

Both surgeons and coordinators recognised, however, that *anticipating outcomes* was not straightforward, with participants reporting a high degree of unpredictability and subjectivity around donor assessment, and noting that real-world clinical outcomes may not align entirely with academic evidence or with hypothetical predictions.

Yes, that [being surprised by an outcome] happens often. I guess it is primary graft dysfunction that we are talking about and we don’t fully have control of that yet. It’s multifactorial and, for heart for example, it might be absolutely fine, no support or anything, but then retrieval happens, further insult, injury during ischaemic time and so on, then the implant and the toxic milieu of the recipient. You can never really guestimate what the outcome of all that will be - Consultant Surgeon 05

The surgeon’s decision to accept an offer of a donated heart or lung was therefore presented as a balanced judgement of perceived risks for a known but unpredictable situation, weighed against a hypothetical future scenario.

The whole of transplant is a risk benefit game of will there be a better donor in future? Will there be a better organ or should we accept this one? - Consultant Surgeon 01

Participants also acknowledged the potential of other non-rational influences on their judgement. Both groups of participants described how, after being involved in cases with a poor recipient outcome, they felt less inclined to take the risk of proceeding when faced with other scenarios with similar characteristics. Many described the enduring impact of being involved in especially ‘horrendous’ cases that ‘stand out in our minds’ and noted how this recalibration of the assessment of risk could override a more rational judgement.

I took a donor with asthma and the recipient had a really horrendous time afterwards. Now I don’t know if it was the asthma or whether it was something else, but actually, for a good period after that, there was a time that I was much more cautious about donors with asthma. Although, you know, the data would suggest that you can quite easily use them - Consultant Surgeon 02

### Data

Central to the process of decision-making and the *anticipation of outcomes* was reviewing and interpreting all available *data* from the donor referral and assessment, as well as recipient *data*. For both coordinators and surgeons, the importance of timely access to an adequate level of detailed, credible and trusted information was strongly emphasised, particularly regarding donor assessment. Participants noted that in practice, lacking access to *data* increased hesitancy to accept the organ.

Often things [information] aren’t available but even [if they are], just sometimes you need more answers around the information. So maybe [the specialist nurses in organ donation] have made a comment about smoking but they haven’t expanded on that. It says ‘smoker’ but smoking what? For how long? When did they stop? - Recipient Coordinator 07

Coordinator participants emphasised the importance of having all the information available to present to their surgeon colleague. Both groups of participants discussed that information sharing between transplant surgeon and coordinator was central to support decision-making.

So, the coordinators field the initial referral, the information from the [donor summary] form will then come to me, plus any imaging. It depends on the experience of the coordinator. Some people will say ‘I’ve looked and we have three people in range [size match]’ and then they will tell me their names. But I still go through the list myself and make sure they haven’t missed anyone - Consultant Surgeon 02

It was also acknowledged that sets of summarised data incorporate interpretations by other individuals, with pertinent details potentially lost or emphasised as the information is collated and curated.

We often try to look at the [donor’s investigation] images, because you can have a good report but a terrible image or vice versa. The information we get on the [donor summary] form is just abstract, so we’ve had issues before where we have missed things from the full [investigation] report, the information on the [donor summary] form looks good but when you look at the full [investigation] report there’s a problem. So, we’ve changed the decision based on those things - Recipient Coordinator 03

Participants discussed that donor organ assessment (in a distant donor hospital, by a potentially unknown retrieval surgeon and team) carried a high level of subjectivity. Consequently, participants recognised that their level of confidence in the information provided varied, depending on social factors such as team dynamics and interpersonal relationships. This links to the theme of *microculture*, as participants described considering the reliability of the donor assessment in view of who was making it, preferentially trusting information from familiar sources.

The good thing was, it was our team retrieving, so we had a better idea of what was going on, a better understanding of what we were dealing with. Obviously, if it was a different team, we would have been a bit more careful, but our team, you know much better the assessment that they can do. So, it was helpful that it was our team and we managed to use that donor and get a heart for our recipient. A heart that would have been rejected otherwise. It was rejected by everyone, except us. And it worked well - Consultant Surgeon 04

Participants recognised the risk of ‘biased’ accounts, when the information and clinical judgements regarding a specific case could be deliberately framed to attempt to guide a colleague. This was described predominantly as coordinator participants delivering, and surgeon participants receiving, information that had been tailored in such a way, to both persuade and dissuade surgeon colleagues to accept a specific organ offer.

I think we have to be honest, I’m no different, [no] less biased. I’ll look at something, I’ll decide if I’m interested and then sell that. We do. We try not to, but you probably do - Recipient Coordinator 01

### Motivation

All participants alluded to a *motivation*, a form of intrinsic ‘drive’, that framed a default attitude towards accepting an offer of an organ. Coordinator participants especially reported that their personal relationships with prospective recipients could translate into the act of advocating for them, seeking to effectively lower the threshold for surgeons to accept an offer made for them. The influence of ‘knowing your patients’ links the theme of *motivation* to the theme of *anticipation of outcomes*.

That [wanting to get someone transplanted] is a very real feeling. I met a lot of the urgent hearts that were [hospital in-patients], you would spend quite a bit of time with them, answering their questions, because they are in the hospital for a long time. And it’s been a lot of people from [home town] and I’ve developed a bit of a bond with the, and, you know, it is a real thing feeling like, we’ve got to do this, we’ve got to get them transplanted, a real drive to do it - Recipient Coordinator 06

While surgeon participants noted this instinct too, they also recognised a more inward-facing driver in the form of a personal ‘passion’ or challenge. This was more evident in the accounts from surgeons with more years of experience.

The only incentive to do transplant these days is your passion. If you don’t have that, I can’t see anybody really interested in transplant these days - Consultant Surgeon 05

It was noted that this default *motivation* to transplant could be attenuated, however, by fatigue, for example, an aspect that links to the *out-of-hours* theme.

It also depends on mood. How people are feeling, how much sleep they have had the night before and, because sometimes, you can pick up the phone to discuss an offer and it’s just ‘no’. In the first three seconds. You know, you barely get to the offer and they have already declined it. Straight away. So I just think sometimes it depends on their mood - Recipient Coordinator 06

### Microculture

Both groups of participants recognised that organ utilisation decisions occurred in the context of the wider department. As a result, decisions were influenced by a working *microculture*, encompassing both clinicians’ interpersonal relationships and the perceived social norms of the department. Both surgeon and coordinator participants discussed that the implanting surgeon is accountable for the ‘final decision’.

The only person who can make the final decision is the implanting surgeon. At the end of the day, it’s their responsibility, for the recipient. The donor is already dead. The final responsibility is between the implant surgeon and the recipient - Consultant Surgeon 01

However, participants explained that in practice the surgeon’s decision to accept or decline a specific offer involved wider considerations and collaborative discussion, with the surgeon incorporating contributions from clinicians across the multidisciplinary team.

If we get an offer that we think is good, then there’s usually a discussion between the surgeon and ourselves. Sometimes there are others involved and usually the cardiologists as well. We would share [information] and discuss with the cardiologists, so that everyone has had a look before we formally accept an offer - Recipient Coordinator 10

In this context, participants recognised that their own perceptions of acceptable risk were dynamic and could be swayed in response to interactions with other clinicians.

So we have surgeons that are more conservative and then surgeons who are more proactive. I think my decision probably falls in line with who I am on call with more than me fully making my own decision. Because at the end of the day, and I say this a lot, as coordinators, it’s not my decision. I’m not the implanting surgeon. - Recipient Coordinator 07

Participants also described anticipating scrutiny by others of the decision to accept or decline an organ. This included formal discussion of completed decisions at departmental meetings, and, less prevalent in accounts, at national level. Surgeon participants reported being cognisant of how their decisions might later be perceived or appraised by their surgeon colleagues, or the wider team, and reported calibrating their decisions accordingly.

Since we had the regular [review of offer] meeting, things have kind of ‘levelled up’. It’s really interesting actually. I mean, it certainly focusses the mind. The threshold to accept certainly went down once we started that meeting. You feel differently if you are potentially going to be questioned as to why you didn’t accept something - Recipient Coordinator 01

The idea of anticipating how a decision may be viewed also extended to the broader transplantation and clinical community. For example, participants described knowing a specific organ offer had already been declined by another centre ‘naturally’ influenced their own centre’s decision.

Some surgeons will ask if other centres have turned [the offer] down, which I think probably affects their decision making. I think that’s something, especially over the last 6-years, I’ve noticed, more so now I think, than when I started - Recipient Coordinator 07

Participants also recounted being aware of how transplantation is viewed by the wider hospital trust. Participants described how decisions to accept high-risk donors or recipients were moderated if intensive care bed capacity was limited, in anticipation of the response of colleagues working in intensive care.

If we know that we are struggling [for intensive therapy unit capacity] then we would be more cautious, or if we have recipients that have sadly not done so well recently, then we might be more careful, more cautious. That’s the reality, isn’t it? If we have three people on ECMO [extra corporeal membrane oxygenation]) that weren’t expected to go on ECMO, then it’s a worry for our patients and for us as a centre. So when you are looking at an offer, you have that in the back of your mind, this has to be a good offer for us to go forward with it - Recipient Coordinator 09

### Out-of-hours

All participants’ accounts of utilisation decision-making referred to the time of day when it occurred. The fact that decision-making often occurred *out-of-hours,* overnight and in addition to scheduled activity, was considered to give important context to the process. Both groups of participants acknowledged that cognitive abilities and judgement varied in response to fatigue.

It’s very different looking at data in the cold light of day, rather than just being woken up at 3 [in the morning], when someone might not be thinking clearly or have other things to think about - Consultant Surgeon 02

Surgeon participants particularly discussed the implications of working patterns, pertinent when the offer was presented to a surgeon who would not, ultimately, implant the organ.

I see sometimes people are more bullish accepting organs if the organ will be coming the next day, when I’m on [duty]. People seem more keen to accept organs for [more complex recipients] when they know they won’t be doing [the operation]. I think those subjective human factors influence judgement - Consultant Surgeon 01

This is one aspect where surgeon and coordinator participants reported different experiences of working patterns; in contrast to a surgeon’s on-call commitments extending after planned daytime activities, coordinators described working more frequently within a rotating shift-based system.

It depends on the time of day of the offer, so at 2 [in the morning], you know, I’m very aware that our surgeons sometimes, you are ringing them in the middle of the night, they have [an operating] list the next day. So you know, they don’t want to stay on the phone very long - Recipient Coordinator 06

Related to the theme of *microculture*, it was also noted that *out-of-hours* there was reduced access to a full multidisciplinary team with whom difficult decisions could be discussed and shared. Both groups of participants acknowledged the value of sharing the burden of responsibility for decision-making with colleagues, which was impacted by the reduced availability of other members of the team during *out-of-hours* working.

During the night, we don’t tend to go to anyone else. But [during the day] you often have a double consultation, with the physicians - Recipient Coordinator 01

### Experiential learning

During the interviews, participants were asked to consider how they had developed the skills to support or make organ-utilisation decisions. While this was not always easy for participants to articulate, many reflected on how over time they learn to ‘get a feel for an organ or a recipient’ and hone their intuition.

It’s not necessarily science based. Some of it is. Some of it is gut instinct and experience - Consultant Surgeon 01

Both groups of participants noted that they became more confident supporting or making decisions as they accumulated personal and vicarious experience, recognising that risk perception is dynamic and, in line with the theme of *microculture*, influenced by others.

[my decision making] has changed massively. Now I push more. I was more conservative than I am now. I think it is that I am more confident. Also, having seen colleagues, when they choose more marginal donors and you see those patients going through without much problem, that has contributed to it – Consultant Surgeon 03

More junior participants reported some formal learning from others, predominantly through professional relationships and open discussion. Mostly participants emphasised *experiential learning* through immersion in the clinical community, iteratively refining their own intuitive practice through working with others.

[how did you learn to do this?] I don’t know, practice I suppose. You just learn while doing the job. Well you kind of learn. I just think I’ve worked here too long now. I suppose a lot of it is about building relationships with people. That and having lots of open discussions and conversation every day. Having a relationship with the surgeons and the cardiologists and the patients. Fostering that sort of open conversation. I think nurses are particularly good at that, in a way doctors sometimes aren’t. So I think practice and watching other people, seeing how they do things – Recipient Coordinator 10

This aspect of developing decision-making skills relates to the theme of *microculture,* in that clinicians reported that their *experiential learning* was shaped by the setting in which they were practising. Surgeons who had practised or trained in other centres acknowledged different prevailing attitudes towards risk and that individuals tended towards mirroring colleagues and adopting the practices within their current centre.

I was away last year and [in the other centre] it was more like ‘let’s do everything’. Accept everything, take every lung. I came back with that sort of attitude. And it has been a lot more tricky here. I’m not sort of jumping to send a team out to have a look [at the organ at the donor site]. Usually I would send a team, let’s just have a look anyway and then decide. But I’m doing less of that, less pushing the boundaries in terms of marginal organs, I guess – Consultant Surgeon 08

## Discussion

This paper is the first to document the range of factors which shape cardiothoracic’ organ utilisation decision-making in practice. Through the accounts of those who directly make and support organ utilisation decisions we illuminate the ways in which decision-making is shaped by the interplay of multiple determinants and elucidate how decision-making skills are learnt and refined over time. This provides evidence for intuitive perceptions of how organ utilisation decision-making is enacted that reveals a more complicated understanding beyond that acknowledged in formal policy. Our themes can be considered in relation to three levels: (1) individual clinicians, (2) transplant teams and (3) organisational factors. Individual factors include the recalibration of risk judgments in response to recent cases and the capacity to make complex decisions in the context of intensive out-of-hours working. Team-based factors include data-sharing between surgeons and coordinators and preferentially trusting information from certain sources. Organisational factors include variable access to colleagues out-of-hours and hospital capacity. Like our themes, these levels are interrelated, with organisational structures (eg, surgeon working patterns), and team-based factors (eg, the dynamic perception of risk of response to others) exerting an influence on human-level decision-making.

The study has several strengths. The interviews were in-depth and included clinicians from all six cardiothoracic transplant centres in the UK. The primary investigator was a cardiothoracic clinician, undertaking a doctoral fellowship, specifically trained in qualitative interviewing. Familiarity with the clinical context may have enhanced her ability to explore participants’ perspectives in a nuanced and credible way. The methods were robust and methodologically coherent. The practice of iteratively and contemporaneously analysing transcripts alongside data collection enabled newly emerging aspects to be explored. Regarding limitations, qualitative interviews have some inherent methodological limitations. The interview process constructs a retrospective version of events, and participants may not be aware of, or adequately recall, all factors that influence decision-making in real time. It is possible that participants were not candid when interviewed by a clinician-researcher with whom some had professional clinical contact. We considered ways to mitigate this, including informing participants of the aim to explore utilisation decision-making in general terms, and using an interview technique that encouraged circumspection, framing questions toward observations of others’ practice. We cannot exclude the possibility, however, that our findings have foregrounded certain aspects that our participants have chosen to emphasise. These factors mean that observing decision-making in real time would add valuable insights. Although efforts were made to purposively sample across a range of centres and experience, participants were self-selected and included fewer surgeons (9/19) who have ultimate decision-making responsibility. It is possible that other pertinent aspects have not been accounted for, including varied centre or individual workload. Heart and lung offers were also considered together in this work, and participants were limited to cardiothoracic transplant surgeons and coordinators. These types of participants represent the core decision-makers (surgeons) and those who most frequently support utilisation decisions (coordinators) in cardiothoracic transplantation, but these two disciplines only contribute part of overall transplantation care. Interviewing clinicians from other disciplinary backgrounds may provide a more complete picture of collaborative decision-making that may differ between organs.

While there are no directly comparable studies in cardiothoracic transplantation, many of our findings are mirrored in the wider transplantation literature. The centrality of timely access to detailed donor information is recognised in kidney transplantation; clinicians discussing procurement biopsies highlighted the importance of quality *data* but recognised a lack of standardisation and consensus on interpretation.^42^ Our interview participants also discussed inherent uncertainties in the *anticipation of outcomes*. This has been noted too by lung transplant candidates who described transplantation as a balance of risks in the setting of uncertainty^43^ and kidney physicians who describe transplantation as ‘a quagmire of subjectivity, interpretation of risk and uncertainties’.^44^ The influence of familiarity with an individual patient’s circumstances described by our participants is reported too by nephrologists, who acknowledged championing their own patients, particularly those with whom they had a rapport.^44^

Many of the factors influencing decision-making identified in our study have also been explored beyond transplantation. The physiological and cognitive impact of managing unscheduled workloads both day and night is well-recognised in wider clinical settings.^45^ Many cognitive biases recognised across the spectrum of surgical practice^27^ were evident in our data. These include the tendency towards action rather than inaction (commission bias, related to our theme of *motivation*), the impact of recent cases with undesirable outcomes (variation in risk aversion) and a form of ambiguity aversion in the tendency to overvalue data from known sources (eg, retrieval teams). In vascular surgery, interviews with interventionalists provided evidence that clinicians’ value judgements are frequently incorporated into the decision-making process.^46^ The importance of seeking to ‘do the right thing for patients’ that was central to participants’ accounts is related to the concept of phronesis in clinical education. Phronesis is the practical wisdom to deliver healthcare in the best way possible and there is wider work to understand how clinicians are trained to incorporate moral skills and virtues into decision-making.^47^ Our findings emphasise the importance of understanding the settings and processes that contribute to *experiential learning* related to organ utilisation decision-making. Future work is needed to explore how best to support clinicians to develop the moral skills and virtues—alongside the clinical knowledge—necessary to make organ utilisation decisions. The themes of *microculture* and *experiential learning* explored participants’ sensitivity to normative patterns of behaviour by their departmental colleagues, adopting the prevailing risk appetite of their centre and calibrating decisions by anticipating retrospective scrutiny by peers. This has been noted in other surgical settings too. General surgeons’ decision-making was affected by their training location and years of experience.^48^ In breast surgery individual surgeons exhibit strong personal preferences for different techniques depending on their experiences and clinical environment.^49^ The same study attributed between-centre variation in part to different volumes of surgical activity and differences in operation scheduling, aspects pertinent too in cardiothoracic transplantation.

Although participants in these interviews referred to a form of distributed decision-making, this contrasts with the scheduled formalised multidisciplinary team (MDT) decision-making meeting structures, like those in cancer settings.^50^ Organ utilisation decision-making is a more individual process, in part reflecting the time-pressures inherent in transplantation. There was notably little discussion from the participants in this study regarding shared decision-making involving the prospective recipient. This mirrors findings from a kidney setting where clinicians reported accepting or declining an organ offer was a medical decision with little room for patient participation.^51^ Involving prospective recipients in decision-making requires more than formalised MDT working however, as shown in research in other settings.^52^

Our participants’ data show that organ utilisation decisions are not being made purely rationally in the context of an objective evidence base. Instead, the impact on decision-making of individuals’ vulnerabilities and inconsistencies influenced by the wider context were confirmed. This aligns with wider work, with Scantlebury *et al*^28^ recently conceptualising decision-making in orthopaedic surgery as a kind of complex intervention; a ‘constant process of medical brokering’ that occurs in a dynamic and fluid way. The authors propose three decision-making ‘linchpins’ (surgeon identity, organisational capacity and socialisation) and discuss how interconnected drivers of decision-making are differentially weighted in real time for each surgical decision depending on the context. Arguably, organ transplantation is even more complex than orthopaedics, as the implications of an organ utilisation decision extend beyond an individual patient to the pool of prospective recipients. Rather than representing a set of hierarchical factors that influence a clinical decision, our data resonate with a sophisticated understanding that decision-making involves balancing a ‘competing set of inter-woven, context-dependent drivers of decision-making situated in a complex evidence economy’.^28^ Our findings indicate that good quality *data* is considered central to supporting the decision to accept or decline an organ, but that this *data* is both presented and interpreted in a way that is subject to human influence. This reveals a potential that the chances of an individual receiving a transplant may fluctuate in ways that are difficult to describe. In solid organ transplantation, there is emerging interest in the use of artificial intelligence-driven tools and decision aids to support clinicians’ organ utilisation decision-making.^53 54^ This work is designed in part to mitigate the risk of inconsistent decision-making by human actors vulnerable to cognitive biases interpreting curated information—and is a developing area of focus.

This is the first time that qualitative data exploring the complexity of interacting factors influencing clinicians’ organ utilisation decision-making has been gathered. We provide documented evidence for hypotheses and experiential assertions and draw attention to the human factors at play in organ utilisation decision-making. Our findings invite a timely discussion of decision-making in solid organ transplantation more widely. There are potential implications for proposed changes to organisation practice in transplantation, including mandatory reviews of declined organ offers, national oversight of educational interventions, initiatives to support daytime organ implantation^55^ and the implementation of organ assessment and recovery centres.^10^ In addition, there are advances in technologies and interventions designed to improve organ availability, including donor optimisation,^56^ normothermic regional perfusion,^57^ ex situ machine perfusion^58 59^ and machine learning techniques as clinical decision aids.^53 54^ Our data suggest that there are vulnerabilities and inconsistencies of the human actors in the transplantation pathway and signal a need for future research into the implications for the uptake of novel tools.

## Conclusion

There is increasing focus on improving the utilisation of organs offered for transplantation, but to date an incomplete understanding of the human factors influencing clinicians’ decision-making. This paper uses qualitative interviews to explore how cardiothoracic transplant surgeons and coordinators approach the decision to accept or decline offers of donated organs. It provides novel empirical data that documents the varied and interactive determinants of organ utilisation decision-making in practice. These data provide evidence of the complexity of real-world decision-making in organ utilisation. This understanding has potential to inform strategies to improve organ utilisation in solid organ transplantation.

## Supplementary material

10.1136/bmjopen-2026-121139 online supplemental file 1

## Data Availability

Data are available upon reasonable request.

## References

[1] Watson CJE, Dark JH (2012). Organ transplantation: historical perspective and current practice. Br J Anaesth.

[2] Black CK, Termanini KM, Aguirre O (2018). Solid organ transplantation in the 21st century. Ann Transl Med.

[3] NHS Blood and Transplant (2024). Organ and tissue donation and transplantation activity report 2023/2024. https://nhsbtdbe.blob.core.windows.net/umbraco-assets-corp/33778/activity-report-2023-2024.pdf.

[4] Smith M, Murphy P (2008). The Ministerial Taskforce on Organ Donation. Journal of the Intensive Care Society.

[5] Schroder JN, Patel CB, DeVore AD (2023). Transplantation Outcomes with Donor Hearts after Circulatory Death. N Engl J Med.

[6] Prieto D, Correia P, Baptista M (2015). Outcome after heart transplantation from older donor age: expanding the donor pool. Eur J Cardiothorac Surg.

[7] Bonser RS, Taylor R, Collett D (2012). Effect of donor smoking on survival after lung transplantation: a cohort study of a prospective registry. The Lancet.

[8] Hardman G, Hogg R, Rushton S (2020). Expanding Donor Age Criteria: The UK Experience. The Journal of Heart and Lung Transplantation.

[9] NHS Blood and Transplant (2025). Annual report on organ utilisation: report for 2024/2025. https://nhsbtdbe.blob.core.windows.net/umbraco-assets-corp/37330/organ-utilisation-annual-report-2024-25-final.pdf.

[10] Department of Health and Social Care (2023). CP 793 – honouring the gift of donation: utilising organs for transplant – report of the organ utilisation group – february 2023. https://assets.publishing.service.gov.uk/media/63ee123b8fa8f5612c4f5316/Honouring-the-gift-of-donation-utilising-organs-for-transport-OUG-report-print-ready.pdf.

[11] Neuberger J, Callaghan C (2020). Organ utilization - the next hurdle in transplantation?. Transpl Int.

[12] Williment C, Jones J, Forsythe J (2023). Excellence in Organ Utilisation-A Quantitative and Qualitative Evidence Base for a New Approach in the UK. Transpl Int.

[13] Martin F, Carmona M, Mahillo B (2026). Organ Donation and Transplantation Worldwide: The Global Observatory on Donation and Transplantation 2024 Report. Transplantation.

[14] Green E, Dutcher EG, Schold JD (2025). The dynamics of deceased donor kidney transplant decision making: insights from studying individual clinicians’ offer decisions. Am J Transplant.

[15] Greenberg JW, Fatuzzo SH, Ramineni A (2023). Heart transplant offers are less likely to be accepted on weekends, holidays, and conferences. J Heart Lung Transplant.

[16] Summers DM, Ayorinde JOO, Neil DA (2025). Evaluation of a national digital pre-implantation biopsy service for deceased-donor kidney transplantation in the UK (Pithia trial); a stepped-wedge cluster randomised registry trial. Lancet Reg Health Eur.

[17] Vinson AJ, Cardinal H, Parsons C (2023). Disparities in Deceased Donor Kidney Offer Acceptance: A Survey of Canadian Transplant Nephrologists, General Surgeons and Urologists. Can J Kidney Health Dis.

[18] Volk ML, Roney M, Merion RM (2013). Systematic bias in surgeons’ predictions of the donor-specific risk of liver transplant graft failure. Liver Transpl.

[19] Department of Health and Social Care (2025). Cardiothoracic information collation exercise: patient and staff survey report. https://www.gov.uk/government/publications/cardiothoracic-transplant-information-collation-exercise-survey-analysis/cardiothoracic-information-collation-exercise-patient-and-staff-survey-report.

[20] BBC News (2026). UK’s transplant system was world-leading - now it lags behind other western nations. https://www.bbc.co.uk/news/articles/clyrj8rz6jno.

[21] NHS Blood and Transplant (2024). Lung candidate selection criteria. https://nhsbtdbe.blob.core.windows.net/umbraco-assets-corp/35163/pol231.pdf.

[22] NHS Blood and Transplant (2024). Heart transplantation: selection criteria and recipient registration. https://nhsbtdbe.blob.core.windows.net/umbraco-assets-corp/34323/pol229.pdf.

[23] NHS Blood and Transplant (2024). Heart transplant organ allocation policy. https://nhsbtdbe.blob.core.windows.net/umbraco-assets-corp/34520/pol228-heart-transplantation-organ-allocation.pdf.

[24] NHS Blood and Transplant (2024). Donor lung distribution and allocation policy. https://nhsbtdbe.blob.core.windows.net/umbraco-assets-corp/34575/pol230.pdf.

[25] Chilvers N, Dark JH (2023). Heart transplantation. Surgery (Oxford).

[26] Andreasson ASI, Dark JH (2023). Lung transplantation: state of the art and current practice. Surgery (Oxford).

[27] Armstrong BA, Dutescu IA, Tung A (2023). Cognitive biases in surgery: systematic review. Br J Surg.

[28] Scantlebury A, Jones K, Adamson J (2025). Can we ever have evidence-based decision making in orthopaedics? A qualitative evidence synthesis and conceptual framework. BMC Med Inform Decis Mak.

[29] White SL (2017). Nudging the Organ Discard Problem. Transplantation.

[30] Nadaf H, Jabade M, Khande A (2025). Exploring the Dynamics: A Comprehensive Scoping Review of Factors Influencing Organ Donation Decisions: A Scoping Review. Iran J Public Health.

[31] Grossi AA (2025). Shared Decision-Making in Solid Organ Transplantation: A Review. Transplantology.

[32] Heilman RL, Green EP, Reddy KS (2017). Potential Impact of Risk and Loss Aversion on the Process of Accepting Kidneys for Transplantation. Transplantation.

[33] Carminati L (2020). Behavioural Economics and Human Decision Making: Instances from the Health Care System. Health Policy.

[34] Butler A, Chapman G, Johnson JN (2020). Behavioral economics—A framework for donor organ decision‐making in pediatric heart transplantation. Pediatr Transplant.

[35] Sharif A (2022). Risk Aversion, Organ Utilization and Changing Behavior. Transpl Int.

[36] Tong A, Morton RL, Webster AC (2016). How Qualitative Research Informs Clinical and Policy Decision Making in Transplantation: A Review. Transplantation.

[37] Charmaz K (2024). Constructing Grounded Theory.

[38] Madill A, Jordan A, Shirley C (2000). Objectivity and reliability in qualitative analysis: realist, contextualist and radical constructionist epistemologies. Br J Psychol.

[39] Tong A, Sainsbury P, Craig J (2007). Consolidated criteria for reporting qualitative research (COREQ): a 32-item checklist for interviews and focus groups. Int J Qual Health Care.

[40] NHS Blood and Transplant (2025). Organ donation and transplantation clinical: cardiothoracic advisory group. https://www.odt.nhs.uk/transplantation/cardiothoracic/cardiothoracic-advisory-group/.

[41] Vasileiou K, Barnett J, Thorpe S (2018). Characterising and justifying sample size sufficiency in interview-based studies: systematic analysis of qualitative health research over a 15-year period. BMC Med Res Methodol.

[42] Schantz K, Gordon EJ, Lee U (2022). The Role of Procurement Biopsies in Kidney Acceptance Decision Making and Kidney Discard: Perceptions of Physicians, Nurse Coordinators, and OPO Staff and Directors. Transplant Direct.

[43] Chen M, Zou X, Nan J (2023). Patient Experiences and Perspectives of Their Decision-Making to Accept Lung Transplantation Referral: A Qualitative Study. IJERPH.

[44] Tong A, Howard K, Wong G (2011). Nephrologists’ perspectives on waitlisting and allocation of deceased donor kidneys for transplant. Am J Kidney Dis.

[45] Agyapong-Opoku F, Agyapong-Opoku N, Agyapong B (2025). Examining the Effects of Sleep Deprivation on Decision-Making: A Scoping Review. Behav Sci (Basel).

[46] Powell CA, Szczygiel LA, Byrnes ME (2026). A qualitative investigation of interventionalists’ views and experiences on clinical decision-making in claudication. JVS-Vascular Insights.

[47] Plews-Ogan M, Sharpe KE (2022). Phronesis in Medical Practice: The Will and the Skill Needed to Do the Right Thing. Springer Nature Singapore.

[48] Gunaratnam C, Bernstein M (2018). Factors Affecting Surgical Decision-making-A Qualitative Study. Rambam Maimonides Med J.

[49] Yun JY, Jeon DN, Jeon B-J (2024). Factors influencing the decision-making process in breast reconstruction from the perspective of reconstructive surgeons: A qualitative study involving Korean plastic surgeons. Journal of Plastic, Reconstructive & Aesthetic Surgery.

[50] Rosell L, Alexandersson N, Hagberg O (2018). Benefits, barriers and opinions on multidisciplinary team meetings: a survey in Swedish cancer care. BMC Health Serv Res.

[51] Cardinal H, Ballesteros Gallego F, Affdal A (2020). Canadian transplant nephrologists’ perspectives on the decision-making process for accepting or refusing a kidney from a deceased organ donor. Clin Transplant.

[52] Hamilton DW, Heaven B, Thomson R (2022). How do patients make decisions in the context of a multidisciplinary team: an ethnographic study of four head and neck cancer centres in the north of England. BMJ Open.

[53] Kourounis G, Elmahmudi AA, Thomson B (2025). Deep learning for automated boundary detection and segmentation in organ donation photography. Innov Surg Sci.

[54] Gotlieb N, Azhie A, Sharma D (2022). The promise of machine learning applications in solid organ transplantation. NPJ Digit Med.

[55] NHS Blood and Transplant (2025). Sustainability and certainty in organ retrieval (score). https://www.odt.nhs.uk/retrieval/sustainability-and-certainty-in-organ-retrieval-score/.

[56] Bera KD, Shah A, English MR (2020). Optimisation of the organ donor and effects on transplanted organs: a narrative review on current practice and future directions. Anaesthesia.

[57] Oniscu GC, Mehew J, Butler AJ (2023). Improved Organ Utilization and Better Transplant Outcomes With In Situ Normothermic Regional Perfusion in Controlled Donation After Circulatory Death. Transplantation.

[58] Tingle SJ, Dobbins JJ, Thompson ER (2023). Machine perfusion in liver transplantation. Cochrane Database Syst Rev.

[59] Tingle SJ, Thompson ER, Figueiredo RS (2024). Normothermic and hypothermic machine perfusion preservation versus static cold storage for deceased donor kidney transplantation. Cochrane Database Syst Rev.

