# Supplementary material for: “It’s not necessarily science based”: understanding organ utilisation decision-making in cardiothoracic transplantation in the UK through qualitative interviews
Source: BMJ Open. 2026 Oct 5;16(10):e121139. doi: 10.1136/bmjopen-2026-121139 (PMC13646951; doi:10.1136/bmjopen-2026-121139)
Supplement: Supplementary file 1 [file bmjopen-2026-121139.reviewer_comments.pdf]

# PEER REVIEW HISTORY

BMJ Open publishes all reviews undertaken for accepted manuscripts. Reviewers are asked to complete a checklist review form and are provided with free text boxes to elaborate on their assessment. These free text comments are reproduced below.

## ARTICLE DETAILS

### Title (Provisional)

“It’s not necessarily science based” - Understanding organ utilisation decision-making in cardiothoracic transplantation in the UK through qualitative interviews

### Authors

Jenkins, Rebeka; Hardman, Gillian; Booth, Karen; Dark, John; Fisher, Andrew; Exley, Catherine

---

## VERSION 1 - REVIEW

---

| | |
|---|---|
| **Reviewer** | **1** |
| **Name** | **Gibbons, Andrea** |
| **Affiliation** | **University of Winchester Department of Psychology** |
| **Date** | **01-Jun-2026** |
| **COI** | **None** |

---

This qualitative paper explores decision making in cardiothoracic transplantation.

I think this is a well-written paper of a well-designed study. The analysis is appropriate, the themes are well-supported with quotations, and there are appropriate discussion of the findings to the literature. I also think this an important topic as not enough has been done on this type of decision making in transplantation.

The only (minor) suggestions I would make are:

I’m not sure that there is a clear enough rationale for why grounded theory was used. It would be good to add in a sentence or so that gives more reasoning for the choice of grounded theory over other methodologies/analyses.

It is good that the authors haven’t attempted to make any broad suggestions for changes to processed based on this study, as it is a small qualitative study and should not be used to make generalisations. However I was surprised that experiential learning wasn’t mentioned more in the discussion. I think it would be useful to add in a sentence that focuses on the importance of experiential learning, and that perhaps further work is needed to explore how this could be used in the future to help others feel more confident in their decision making.

| | |
|---|---|
| **Reviewer** | **2** |
| **Name** | **Plumb, Lucy** |
| **Affiliation** | **University of Bristol Medical School** |
| **Date** | **08-Jul-2026** |
| **COI** | **None** |

Thank you for the opportunity to review this interesting study, examining the decision-making processes of healthcare professionals involved in the decision-making process of cardiothoracic organ utilisation. The paper is well written and easy to follow and addresses an important research question. However, I would recommend major revisions based on the following issues raised:

1. My main concern with this paper is that, as it is currently written, it draws the reader to believe that transplant coordinators are decision-makers in the organ utilisation process. My experience and understanding is that, while they may heavily support this process, they are not involved in the final acceptance or decline decision. As understanding the decision-making process is the main aim of the study, I feel it is necessary to re-word the manuscript to make clear that professionals involved in this process were interviewed but that the final decision rests with the transplant surgeon. More clarity in the introduction about who the final decision rests with would be helpful. Also recognising that the main decision-makers are limited to 9/19 sample would be helpful in the limitations and in understanding what can be inferred from the interviews (page 7, line 10 infers all participants are decision-makers, for example). If I am misinformed about the responsibility of the transplant coordinator to make clinical decisions, please ignore this. However, on page 17, recipient coordinator 07's quote suggests this is the case. I therefore think this manuscript needs to reframed for clarity and to present the transplant coordinator data with this in mind.

2. Page 8, lines 52-56: It would be helpful, with the above comment in mind, to understand which participants (transplant coordinator or surgeon) had line-by-line coding completed, as this will influence the nature of future questioning.

3. I am not clear, based on the COREQ questionnaire completed, whether information power or data saturation was used to determine the final sample size of this study. There is limited information in the methods/page 5 as the questionnaire states. Please can the authors elaborate on this.

4. The authors mention the value of information in making a clinical decision but also the potential biases in accepting certain information more readily, triangulated with transplant coordinators suggesting they do select information to 'sell' the case. This appears to be an inherent problem in human oversight of organ utilisation and could, in theory, be minimised with AI/Machine learning-led algorithms to assess organ quality. Can the authors elaborate

on this finding in the discussion and it's clinical implications? There is mention of cognitive biases (page 23, line 57) in terms of more readily accepting information from certain teams but no mention of this (interesting) finding that that information may be selectively presented which influence/aggravate these biases. As the authors comment, there are 'vulnerabilities and inconsistencies of the human actors' and this finding reflects that.

---

## VERSION 1 - AUTHOR RESPONSE

<table>
<tr><td>Comments</td><td>Response</td></tr>
<tr><td colspan="2">Reviewer 1 comments</td></tr>
<tr><td>1. I'm not sure that there is a clear enough rationale for why grounded theory was used. It would be good to add in a sentence or so that gives more reasoning for the choice of grounded theory over other methodologies/analyses.</td><td>The following sentence added to "Study design" section of methods:<br><br>"This supported an inductive interpretivist approach to analysis where there is little existing evidence, allowed for multiple perspectives to be simultaneously acknowledged and for emerging lines of analytical inquiry to be explored in future interviews."</td></tr>
<tr><td>2. It is good that the authors haven't attempted to make any broad suggestions for changes to processed based on this study, as it is a small qualitative study and should not be used to make generalisations. However I was surprised that experiential learning wasn't mentioned more in the discussion. I think it would be useful to add in a sentence that focuses on the importance of experiential learning, and that perhaps further work is needed to explore how this could be used in the future to help others feel more confident in their decision making.</td><td>Thank you for this insight. The following has been added to the 4th paragraph of the Discussion to reflect the recommendation:<br><br>"Our findings emphasise the importance of understanding the settings and processes that contribute to experiential learning related to organ utilisation decision-making. Future work is needed to explore how best to support clinicians to develop the moral skills and virtues - alongside the clinical knowledge - necessary to make organ utilisation decisions."</td></tr>
<tr><td>Reviewer 2 comments</td><td></td></tr>
<tr><td>3. My main concern with this paper is that, as it is currently written, it draws the reader to believe that transplant coordinators are decision-makers in the organ utilisation process. My experience and understanding is that, while they may heavily support this process, they are not involved in the final acceptance or decline decision. As understanding the decision-making process is the main aim of the study, I feel it is necessary to re-word the</td><td>Thank you for this important observation. We agree it is critical to emphasise that final decision-making lies with the transplanting surgeon. The manuscript has been reworded throughout as recommended:<ul><li>Strengths and limitations<ul><li>1st bullet point now reads "Interviews were conducted with the clinicians most closely involved in organ utilisation decisions in heart and lung transplantation"</li></ul></li><li>Introduction</li></ul></td></tr>
</table>

| | |
|---|---|
| manuscript to make clear that professionals involved in this process were interviewed but that the final decision rests with the transplant surgeon.<br><br>More clarity in the introduction about who the final decision rests with would be helpful.<br><br>Also recognising that the main decision-makers are limited to 9/19 sample would be helpful in the limitations and in understanding what can be inferred from the interviews (page 7, line 10 infers all participants are decision-makers, for example). If I am misinformed about the responsibility of the transplant coordinator to make clinical decisions, please ignore this. However, on page 17, recipient coordinator 07's quote suggests this is the case. I therefore think this manuscript needs to reframed for clarity and to present the transplant coordinator data with this in mind. | ○ 3rd paragraph now reads "the decision to accept an organ offered for transplantation rests with the individual transplanting team and specifically with the transplanting surgeon. In the UK, specialist trained cardiothoracic recipient nurse coordinators receive details of an organ offer from a centralised service. They then collate the available information regarding the offer, which is then discussed with the on-call consultant cardiothoracic transplant surgeon. It is the surgeon who makes the final decision to accept or decline the offer supported by the coordinator.<br>○ 4th paragraph now reads "A greater understanding of the factors that influence the transplanting surgeon's decision to accept or decline a donor organ is needed – including the supporting role of the transplant coordinator."<br>○ 5th paragraph now reads "By exploring first-hand accounts from clinicians most closely involved in organ utilisation decisions for heart and lungs, this work aims to deepen understanding of how surgeons describe deciding which donor organs to accept for transplantation supported by transplant coordinators, and how decision-making practice is learned and refined over time.<br>• *Results*<br>○ 7th paragraph of 'Anticipation of outcomes' reads "The surgeon's decision to accept an offer of a donated heart or lung…"<br>○ 1st paragraph of 'Data' reads "Participants noted that in practice, lacking access to data increased hesitancy to accept the organ".<br>○ 2nd paragraph of 'Data' reads "Both groups of participants discussed that information sharing between transplant surgeon and coordinator was central to support decision-making".<br>○ 5th paragraph of 'Data' reads "This was described predominantly as coordinator participants delivering, and surgeon participants receiving, information that had been tailored in such a way, to both persuade and dissuade surgeon colleagues to accept a specific organ offer."<br>○ 1st paragraph of 'Motivation' reads "Coordinator participants especially reported that their personal relationships |

with prospective recipients could translate into the act of advocating for them, seeking to effectively lower the threshold for surgeons to accept an offer made for them

- 2nd paragraph of 'Microculture' reads "However, participants explained that in practice the surgeon's decision to accept or decline a specific offer involved wider considerations and collaborative discussion."
- 4th paragraph of 'Microculture' reads "Participants also described anticipating scrutiny by others of the decision to accept or decline an organ."
- 5th paragraph of 'Microculture' reads "For example, participants described knowing a specific organ offer had already been declined by another centre "naturally" influenced their own centre's decision."
- 6th paragraph of 'Microculture' reads "Participants described how decisions to accept high risk donors or recipients were moderated if intensive care bed capacity was limited, in anticipation of response of colleagues working in intensive care."
- 1st paragraph of 'Out-of-hours' reads "All participants' accounts of ~~their~~ utilisation decision-making referred to the time of day when it occurred"
- 3rd paragraph of 'out-of-hours' reads "This is one aspect where surgeon and coordinator participants reported different experiences of working patterns…"
- 1st paragraph of 'Experiential learning' reads "During the interviews, participants were asked to consider how they had developed the skills to support or make organ-utilisation decisions. Whilst this was not always easy for participants to articulate, many reflected on how over time they learn to "get a feel for an organ or a recipient" and hone their intuition.
- 2nd paragraph of 'Experiential learning' reads "Both groups of participants noted that they became more confident supporting or making decisions…"

* *Discussion*
  - 1st paragraph reads "This paper is the first to document the range of factors which shape cardiothoracic ~~clinicians~~' organ utilisation decision-making in practice. Through the

| | |
|---|---|
| | accounts of those who directly make and support organ utilisation decisions, we illuminate the ways in which decision-making is shaped by the interplay of multiple determinants and elucidate how decision-making skills are learned and refined over time."<br>o 1st paragraph also includes "Team-based factors include data-sharing between surgeons and coordinators..."<br>o 2nd paragraph discussing limitations includes "Although efforts were made to purposively sample across a range of centres and experience, participants were self-selected and included slightly fewer surgeons (9/19) who have ultimate decision-making responsibility."<br>o 2nd paragraph also includes "Heart and lung offers were considered together in this work, and participants were limited to cardiothoracic transplant surgeons and coordinators. These two types of participants represent the core decision-makers (surgeons) and those who most frequently support utilisation decisions (coordinators) in cardiothoracic transplantation, but these two disciplines only contribute part of overall transplantation care.<br>o 3rd paragraph includes "The themes of micro-culture and experiential learning explored participants' sensitivity to normative patterns of behaviour by their departmental colleagues, adopting the prevailing risk appetite of their centre and calibrating ~~their~~ decisions by anticipating retrospective scrutiny by peers."<br>o 4th paragraph includes "Our participants' data show that organ utilisation decisions are not being made purely rationally in the context of an objective evidence base." |
| 4. Page 8, lines 52-56: It would be helpful, with the above comment in mind, to understand which participants (transplant coordinator or surgeon) had line-by-line coding completed, as this will influence the nature of future questioning. | This detail has been added to methods sections as follows:<br>"Line-by-line inductive coding of the first five anonymised transcripts (1 surgeon, 4 coordinators) identified, described and analysed patterns derived directly from the interviews." |
| 5. I am not clear, based on the COREQ questionnaire completed, whether information power or data saturation was used to determine the final sample | Clarifying detail has been added to the methods section as follows: |

| | |
|---|---|
| size of this study. There is limited information in the methods/page 5 as the questionnaire states. Please can the authors elaborate on this. | "Through iterative rounds of analysis more defined themes were developed, latterly using a deductive approach to test and refine themes. Rather than a traditional assessment of data saturation, it was judged that sufficiently rich data from experts with direct lived experience (high information power) had been collected to answer the study's specific research question after 19 interviews - whereafter data collection ceased. |
| 6. The authors mention the value of information in making a clinical decision but also the potential biases in accepting certain information more readily, triangulated with transplant coordinators suggesting they do select information to 'sell' the case. This appears to be an inherent problem in human oversight of organ utilisation and could, in theory, be minimised with AI/Machine learning-led algorithms to assess organ quality. Can the authors elaborate on this finding in the discussion and it's clinical implications? There is mention of cognitive biases (page 23, line 57) in terms of more readily accepting information from certain teams but no mention of this (interesting) finding that that information may be selectively presented which influence/aggravate these biases. As the authors comment, there are 'vulnerabilities and inconsistencies of the human actors' and this finding reflects that. | Thank you for this perceptive observation and the encouragement to elaborate in the discussion.<br><br>Added to 4th paragraph of discussion:<br><br>"Our findings indicate that good quality data is considered central to supporting the decision to accept or decline an organ, but that this data is both presented and interpreted in a way that is subject to human influence. This reveals a potential that the chances of an individual receiving a transplant may fluctuate in ways that are difficult to describe. In solid organ transplantation, there is emerging interest in the use of artificial intelligence-driven tools and decision aids to support clinicians' organ utilisation decision-making 53,54. This work is designed in part to mitigate the risk of inconsistent decision-making by human actors vulnerable to cognitive biases interpreting curated information - and is a developing area of focus." |

## VERSION 2 - REVIEW

| | |
|---|---|
| **Reviewer** | **2** |
| **Name** | **Plumb, Lucy** |
| **Affiliation** | **University of Bristol Medical School** |
| **Date** | **27-Jul-2026** |
| **COI** | |

This revision has appropriately addressed my concerns. I would suggest a couple of minor changes:

1. Please remove 'slightly' or rephrase when referring to the number of surgeons versus transplant coordinators in the limitations (p19). There are simply fewer of the decision-makers who have been sampled.

2. The authors repeatedly suggest in the discussion that this is novel emperic data that is one of a kind. It is mentioned three times. I would suggest that at least one reference could be removed (page 18, lines 51/53, as there is no qualification as to what this is in reference to). I would suggest keeping the justification on page 27, lines 13-16 but dropping the words 'the first of its kind in the UK' on the final page/conclusion (p24).

3. I would suggest the authors rephrase 'this paper' to 'this study' in the objectives of the abstract.

---

## VERSION 2 - AUTHOR RESPONSE

| Comments | Response |
|---|---|
| *Reviewer 2 comments* | |
| 1. Please remove 'slightly' or rephrase when referring to the number of surgeons versus transplant coordinators in the limitations (p19). There are simply fewer of the decision-makers who have been sampled. | Wording has been revised to this paragraph 2 of discussion as recommended.<br>"Although efforts were made to purposively sample across a range of centres and experience, participants were self-selected and included ~~slightly~~ fewer surgeons (9/19) who have ultimate decision-making responsibility." |
| 2. The authors repeatedly suggest in the discussion that this is novel emperic data that is one of a kind. It is mentioned three times. I would suggest that at least one reference could be removed (page 18, lines 51/53, as there is no qualification as to what this is in reference to). I would suggest keeping the justification on page 27, lines 13-16 but dropping the words 'the first of its kind in the UK' on the final page/conclusion (p24). | Discussion paragraph 2 has been amended as suggested:<br>"The study has several strengths. ~~We present novel empirical data that are the first of their kind.~~ The interviews were in-depth and included clinicians from all six cardiothoracic transplant centres in the UK."<br>Conclusion has been amended as suggested:<br>"It provides novel empirical data, ~~the first of its kind in the UK~~, that documents the varied and interactive determinants of organ utilisation decision-making in practice." |
| 3. I would suggest the authors rephrase 'this paper' to 'this study' in the objectives of the abstract. | Wording changed as suggested in Abstract:<br>• "Objectives: : This study aims..."<br>• "Conclusion: This study is..." |
