# Supplementary material for: “It’s not necessarily science based”: understanding organ utilisation decision-making in cardiothoracic transplantation in the UK through qualitative interviews
Source: BMJ Open. 2026 Oct 5;16(10):e121139. doi: 10.1136/bmjopen-2026-121139 (PMC13646951; doi:10.1136/bmjopen-2026-121139)
Supplement: Supplementary file 2 [file bmjopen-16-10-s001.docx]

## Supplementary File 1 COREQ (COnsolidated criteria for REporting Qualitative research) checklist

| **Topic** | **Item no.** | **Guide questions/description** | **Reported on Page Number** |
| --- | --- | --- | --- |
| **Domain 1: Research team and reflexivity** | | | |
| *Personal characteristics* | | | |
| Interviewer/facilitator | 1 | Which author(s) conducted the interview or focus group? | 3 |
| Credentials | 2 | What were the researcher’s credentials (e.g. PhD, MD)? | 3 |
| Occupation | 3 | What was their occupation at the time of the study? | 3 |
| Gender | 4 | Was the researcher male or female? | 3 |
| Experience and training | 5 | What experience or training did the researcher have? | 3, 19 |
| *Relationship with participants* | | |  |
| Relationship established | 6 | Was a relationship established prior to study commencement? | 19 |
| Participant knowledge of the interviewer | 7 | What did the participants know about the researcher (e.g. personal goals, reasons for doing the research)? | 3, 19 |
| Interviewer characteristics | 8 | What characteristics were reported about the interviewer/facilitator (e.g. bias, assumptions, interests)? | 3, 19 |
| **Domain 2: Study design** | | | |
| *Theoretical framework* | | | |
| Methodological orientation and theory | 9 | What methodological orientation was stated to underpin the study (e.g. grounded theory, discourse analysis, ethnography, phenomenology, content analysis)? | 3 |
| *Participant selection* | | |  |
| Sampling | 10 | How were participants selected? (e.g. purposive, convenience, consecutive, snowball) | 4 |
| Method of approach | 11 | How were participants approached (e.g. face-to-face, telephone, email)? | 4 |
| Sample size | 12 | How many participants were in the study? | 5 |
| Non-participation | 13 | How many people refused to participate or dropped out? Reasons? | NA |
| *Setting* | | |  |
| Setting of data collection | 14 | Where was the data collected (e.g. home, clinic, remote)? | 4 |
| Presence of non-participants | 15 | Was anyone else present besides the participants and researchers? | 5 |
| Description of sample | 16 | What are the important characteristics of the sample (e.g. demographic data, date)? | 4,5,6 Table 1 |
| *Data collection* | | |  |
| Interview guide | 17 | Were questions, prompts, guides provided by the authors? Was it pilot tested? | 4, Supplementary file II |
| Repeat interviews | 18 | Were repeat interviews carried out? If yes, how many? | 4 |
| Audio/visual recording | 19 | Did the researcher use audio or visual recording to collect the data? | 5 |
| Field notes | 20 | Were field notes made during and/or after the interview or focus group? | 5 |
| Duration | 21 | What was the duration of the interviews or focus groups? | 5 |
| Data saturation | 22 | Was data saturation discussed? | 5 |
| Transcripts returned | 23 | Were transcripts returned to participants for comment and/or correction? | 5 |
| **Domain 3: analysis and findings** | | | |
| *Data analysis* | | | |
| Number of data coders | 24 | How many data coders coded the data? | 5 |
| Description of the coding tree | 25 | Did authors provide a description of the coding tree? | NA |
| Derivation of themes | 26 | Were themes identified in advance or derived from the data? | 5 |
| Software | 27 | What software, if applicable, was used to manage the data? | 5 |
| Participant checking | 28 | Did participants provide feedback on the findings? | 5 |
| *Reporting* | | |  |
| Quotations presented | 29 | Were participant quotations presented to illustrate the themes/findings? Was each quotation identified (e.g. participant number) | 7-18 |
| Data and findings consistent | 30 | Was there consistency between the data presented and the findings? | 6-18 |
| Clarity of major themes | 31 | Were major themes clearly presented in the findings? | 6 |
| Clarity of minor themes | 32 | Is there a description of diverse cases or discussion of minor themes? | 6-18 |

Developed from: Tong A, Sainsbury P, Craig J. Consolidated criteria for reporting qualitative research (COREQ): a 32-item checklist for interviews and focus groups. *International Journal for Quality in Health Care.* 2007. Volume 19, Number 6: pp. 349 - 357

## Supplementary File II: Interview Topic Guide

This guide is to general topics that will covered in the interviews. It will be used flexibly, so that the participant has the opportunity to share information in a way that is most natural to them. How questions are asked and the order in which they will be asked will vary from interview to interview. If a new topic of interest arises in one interview, this may be added to the guide for inclusion in subsequent interviews.

**Surgeon**

1. Talk me through your decisions for the last donor heart/lung offer you received

2. What do you take into consideration when deciding whether or not to accept a donor organ?

i. Individual factors

Donor	*specific clinical details*

Recipient	*specific clinical characteristics*

ii. Organisational factors

The team	who follows you on the on call?

Other team members

Time of day

Logistics	Beds, COVID pressures, elective operating

Do you consider any one of these elements to be 'the most important'?

What do you think makes a good heart or lung donor? Has this changed over time?

3. How to you think this donor decision making compares to other decisions you make in clinical practice?

- intra-operative decisions

- MDT decisions

4. Talk me through your relationship with the coordinators

a. are you aware if they influence your decisions or not?

b. do they give you their opinion on a donor?

c. are you decisions ever affected by which coordinator you are working with?

5. How do you think your decision making compares to that of other colleagues?

a. Do you talk to your colleagues about how they make these decisions?

b. To what extent do you think about what your colleagues would do or say in response to an offer you receive?

Do you think, generally, that most of your colleagues want to transplant? Why, what makes you think that?

6. How did you learn to make these decisions?

a. do you think anyone can learn how to make these decisions?

b. how do you go about teaching juniors/colleagues to make these decisions?

c. how do you think you support colleagues to think about donor decisions?

7. To what extent, and in what ways, do you think your decision making has changed over time?

8. Do you think about risk? Can you describe those thought processes to me?

a. what does 'risk' mean to you when you talk about donor decisions and transplant

b. Do you think you are more or less risk averse than your colleagues? Tell me about that

9. Can you talk me through the ways you think we could help the decision making process when donor organs are

offered?

**Coordinator**

1. Talk me through your decisions for the last donor heart/lung offer you received

2. What do you take into consideration when deciding whether or not to accept a donor organ?

i. Individual factors

Donor	*specific clinical details*

Recipient	*specific clinical characteristics*

Surgeon	*"Surgeon characteristics" - do your decisions change depending on surgeon?*

ii. Organisational factors

The team	who follows you on the on call?

Other team members

Time of day

Logistics	Beds, COVID pressures, elective operating

Do you consider any one of these elements to be 'the most important'?

What do you think makes a good heart or lung donor? Has this changed over time?

3. Talk me through the ways in which this process might differ for different on call surgeons

Are you aware that you 'behave' differently with different people?

What are those differences and why?

4. Do you make your own decision about the donor before speaking to the surgeon?

a. If so, are you aware at what point you make your decision?

b. Is the surgeon aware of your decision?

c. Do you think your decision has any influence on the surgeons decision? In what ways?

5. Do you think surgeons want to transplant?

6. How often does your decision match that of the surgeon?

7. How did you learn to do this?

Are you involved in teaching others to do this job? Can you talk me through that process - how do you teach others to make decisions about organ donors?

8. To what extent, and in what ways, do you think your decision making has changed over time?

9. Is there anything that you think could help with this decision making in the offering process?

Additions/changes to interview guide April/May 2022

1. Talk me through the last time you declined an offer

2. What other things do you think influence your decisions?

i. night and day

ii. staff members

iii. other

3. How do previous experiences impact your decisions?

a. Do poor outcomes effect what you do next time?

4. Are there particular elements of donors that worry you?

5. What happens when you have a bad outcome? - what is the response, how does the department react, do you feel supported?

a. How do your colleagues deal with it when things go wrong?

6. How do you think the culture in your department influences your decision making?

7. How do you think your decision making compares to that of other colleagues?

i. Do you talk to your colleagues about how they make these decisions?

ii. To what extent do you think about what your colleagues would do or say in response to an offer you receive?

iii. Do you think other centres approach things in the same way?

Do you think, generally, that most of your colleagues want to transplant? Why, what makes you think that?

8. How did you learn to make these decisions?

i. do you think anyone can learn how to make these decisions? - what characteristics do you need?

ii. how do you go about teaching juniors/colleagues to make these decisions?

iii. how do you think you support colleagues to think about donor decisions?

9. Do you think about risk? Can you describe those thought processes to me?

i. what does 'risk' mean to you when you talk about donor decisions and transplant

ii. Do you think you are more or less risk averse than your colleagues? Tell me about that

10. How would you feel about removing this decision from your clinical practice?

i. named patient offering

ii. physicians decision making
